# Image Quality Improvement in Deep Learning Image Reconstruction of Head Computed Tomography Examination

**DOI:** 10.3390/tomography9040118

**Published:** 2023-08-09

**Authors:** Michal Pula, Emilia Kucharczyk, Agata Zdanowicz, Maciej Guzinski

**Affiliations:** 1 Lower Silesian Oncology, Pulmonology and Hematology Center, Hirszfelda Square 12, 53-413 Wrocław, Poland; michal.pula97@gmail.com; 2 Faculty of Medicine, Wroclaw Medical University, Ludwika Pasteura 1, 50-367 Wrocław, Poland; emilia.kucharczyk@student.umed.wroc.pl; 3 Department of General Radiology, Interventional Radiology and Neuroradiology, Wroclaw Medical University, Borowska 213, 50-556 Wrocław, Poland; agatazdanowicz@gmail.com

**Keywords:** deep learning reconstruction, deep neural network, head CT, image quality improvement, true fidelity

## Abstract

In this study, we assess image quality in computed tomography scans reconstructed via DLIR (Deep Learning Image Reconstruction) and compare it with iterative reconstruction ASIR-V (Adaptive Statistical Iterative Reconstruction) in CT (computed tomography) scans of the head. The CT scans of 109 patients were subjected to both objective and subjective evaluation of image quality. The objective evaluation was based on the SNR (signal-to-noise ratio) and CNR (contrast-to-noise ratio) of the brain’s gray and white matter. The regions of interest for our study were set in the BGA (basal ganglia area) and PCF (posterior cranial fossa). Simultaneously, a subjective assessment of image quality, based on brain structure visibility, was conducted by experienced radiologists. In the assessed scans, we obtained up to a 54% increase in SNR for gray matter and a 60% increase for white matter using DLIR in comparison to ASIR-V. Moreover, we achieved a CNR increment of 58% in the BGA structures and 50% in the PCF. In the subjective assessment of the obtained images, DLIR had a mean rating score of 2.8, compared to the mean score of 2.6 for ASIR-V images. In conclusion, DLIR shows improved image quality compared to the standard iterative reconstruction of CT images of the head.

## 1. Introduction

Computed tomography has traditionally been regarded as a fundamental diagnostic modality, particularly in the evaluation of head structures. As a result of technological advancements in CT (computed tomography) imaging and the expanding range of clinical indications, there has been a significant rise in the annual utilization of CT scans. Hence, there is increasing demand for more precise imaging, which minimizes the need for additional tests. This becomes particularly crucial in situations where an urgent response is necessary for the patient’s condition [1,2]. CT scans that are performed without the use of intravenous contrast agents have an inherent limitation in differentiating soft tissues with contrast resolution [3,4]. For instance, in the brain parenchyma, the variation in CT attenuation between gray and white matter is typically minor [5,6]. Identifying specific alterations in attenuation between gray and white matter is necessary for detecting certain intracranial pathologies [4,5,6]. The aforementioned limitations pose considerable challenges in the interpretation of non-contrast CT scans of the head, particularly in cases of trauma [7].

The ability to visualize anatomic structures and pathologic features in a CT image is influenced by two critical factors: blur and noise. Higher levels of blurring can reduce the visibility of minor objects or image details, while higher levels of visual noise can reduce the visibility of low-contrast objects [8]. Therefore, it is crucial to develop an optimized protocol that considers these physical principles for a specific clinical study and adjusts to achieve a balance between detail, low noise, and patient exposure. As the physical parameters of devices are modified at the expense of others, the role of image reconstruction becomes increasingly significant [9,10,11].

Recent advancements in imaging technology have spurred the development of novel techniques for processing CT images. Two prominent image reconstruction techniques used in CT are DLIR (Deep Learning Image Reconstruction) and ASiR-V (Adaptive Statistical Iterative Reconstruction). DLIR utilizes advanced machine learning algorithms to reconstruct CT images [6,10,12], whereas ASiR employs statistical models to iteratively refine the image data [7,13,14]. The primary objective of these reconstruction algorithms is to minimize image noise in low-dose CT scans while preserving image quality [6,9,12,15,16]. In clinical practice, the noise reduction capability of Iterative Reconstruction (IR) is often leveraged to reduce radiation dose while maintaining consistent image noise levels [7,11]. However, IR algorithms have limitations, particularly in balancing noise reduction and image texture, as well as overall image quality [5,6,17]. To tackle these challenges, a novel Deep Learning Image Reconstruction (DLIR) algorithm has been introduced. This algorithm utilizes a deep neural network-based reconstruction engine, which aims to overcome the limitations associated with iterative reconstruction algorithms [5,6,7,17].

The purpose of this study is to compare the quality of images obtained using both of the aforementioned techniques in order to establish a gold standard image reconstruction method for CT of the head. In current research, ASiR remains the benchmark reconstruction model in CT. However, given the constant pursuit of reducing radiation doses while maintaining image quality, and the rising number of parameters subject to evaluation, DLIR appears to be the natural successor to iterative reconstruction (IR).

## 2. Materials and Methods

A total of 163 consecutive head CT scans were retrospectively evaluated. The scans were initially carried out for clinical purposes, using a non-contrast imaging technique. We included 109 patients in the study, and the selection of a suitable research group was based on the implementation of exclusion criteria. These were: (1) neurosurgical treatment such as clipping, coiling, drainage, etc. (7 patients); (2) misregistration artifacts, which appear as blurring, streaking, or shading—caused by patient movement during a CT scan (28 patients); (3) mass effect induced by hematoma, edema, tumors, etc. (19 patients). The purpose of the exclusion criteria was to prevent the degradation of image quality resulting from the existence of various disruptive factors.

A Revolution Apex, 256-row scanner (GE Healthcare, Chicago, IL, USA) was used for all of the examinations. The assessments were conducted using the axial scanning method. All patients were in a supine position; the patient’s head was fixed in the dedicated head holder. The tube voltage was 120 kVp; the mA was set to 320; the beam collimation was 80 mm; the slice thickness was 0.625 mm; the scan range covered the area between the foramen magnum and the top of the head; the scan rotation time was 1 s; and the automatic exposure control (AEC) was activated. DLIR reconstruction was obtained using TrueFidelity, a GE Healthcare deep neural network engine trained on FBP images of high quality, and a DLIR of high strength (DLIR-H) was utilized. The newest generation of iterative reconstruction from GE Healthcare, the Adaptive Statistical Iterative Reconstruction V (ASIR-V), in a combination of 50% ASIR-V and 50% of FBP, was utilized for ASIR-V.

### 2.1. Subjective Image Quality

Paired images were reviewed independently by a group of 3 radiologists with 3, 8, and 10 years of experience in neuroradiology, respectively. The readers were blinded to the reconstruction method. Images were displayed in a brain window (80 W, 40 L) on a GE workstation AW server 3.2 ext. 4.0. The reviewers were requested to evaluate the sharpness of the image, the differentiation between gray and white matter, as well as the visibility of the basal ganglia, pons, cerebellum, and ventricular structures. A Likert scale-based rating system was implemented, where images of excellent quality received a rating of 3, good-quality images were rated 2, and images of poor quality but that were still usable for evaluation received a rating of 1. The figures were assessed sequentially by the radiologist with the least experience, followed by the more experienced readers, with the most proficient neuroradiologist granting the final rank. A Fleiss’ Kappa measurement was utilized to evaluate the agreement between reviewers. The assessment of image quality adhered to the European Guidelines on Quality Criteria for Computed Tomography.

### 2.2. Objective Image Quality:

**Figure 1:**
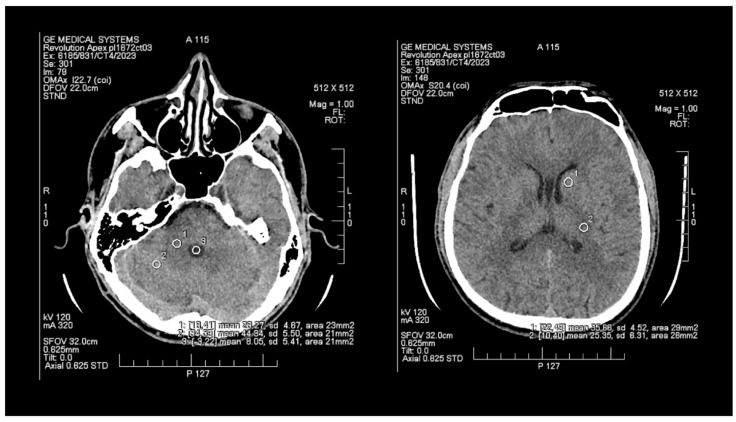
Images of head CT with marked ROIs located in BGA (basal ganglia area) and PCF (posterior cranial fossa) obtained using FBP + ASIR.

**Figure 2:**
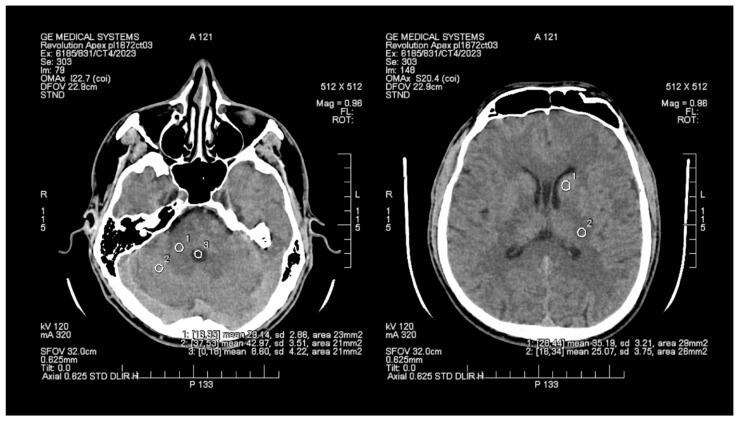
Images of head CT with marked ROIs located in BGA (basal ganglia area) and PCF (posterior cranial fossa) obtained using DLIR.

The objective assessment of image quality involved analyzing the signal-to-noise (SNR) and contrast-to-noise (CNR) ratios. To maintain consistency, a control variable was introduced in the form of manually set Regions of Interest (ROIs), which are analogous and predefined study areas in each pair of images. The average size of the ROI area was 24 mm^2^, with the smallest and the largest areas measuring 20 mm^2^ and 29 mm^2^, respectively. ROIs were positioned manually on the ASIR-V (Figure 1) reconstruction image, and then, duplicated at identical coordinates on the DLIR (Figure 2) reconstruction, which excluded potential deviations resulting from the lack of homogeneity of the measured tissues. The designation of the slice subject to the assessment was based solely on the anatomical factors of the patient. The first two ROIs were located in the white matter of the posterior limb of the internal capsule and the grey matter of the caudate nucleus, while the other two ROIs were positioned in the white and grey matter of the cerebellum. The fifth ROI was set in the cerebrospinal fluid of the fourth ventricle. To determine the signal level and noise, the mean CT number (mean) within the ROI and the standard deviation (SD) were taken into account.

To facilitate data comparison, the calculation of numerical variables was employed, and specific formulae were developed to determine the contrast-to-noise ratio (CNR) and the signal-to-noise ratio (SNR), incorporating the relevant variables. The SNR was calculated by dividing the mean by the standard deviation (SD). For ROI pairs comprising white and grey matter, the CNR was determined using the following formula: the difference between the means of the two ROIs divided by the square root of the sum of the squared noise in the two ROIs.

$$CNR=\frac{Mean1-Mean2}{\sqrt{SD1^{2}+SD2^{2}}}$$

### 2.3. Statistical Analysis

We conducted a statistical analysis using RStudio (version 2022.12.0 + 353) Statistical Software (R Foundation for Statistical Computing, Vienna, Austria) [18]. We performed a paired t-test with Welsh modification for unequal variances to compare the SNR and CNR measurements between image pairs. To reduce the risk of significance due to chance resulting from multiple statistical testing, we applied Bonferroni correction to the *p*-value. A *p*-value of less than 0.01 was considered significant. In order to assess the agreement of the measurements between the standard technique (ASIR) and the novel solution (DLIR), Bland–Altman plots were implemented.

## 3. Results

**Figure 3:**
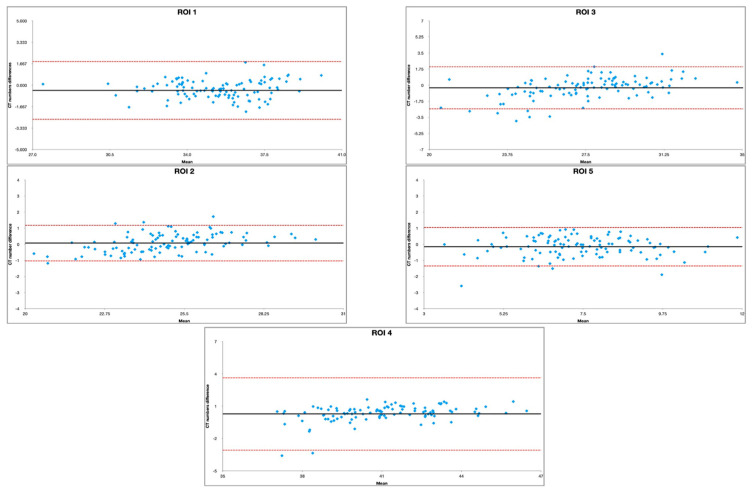
Bland–Altman plot assessment of agreement between DLIR and ASIR in selected ROIs.

The characteristics observed in the Bland–Altman plots for the selected ROIs, namely, uniform dispersion of data points, data points primarily falling within the limits of agreement, no systematic outliners, and a lack of patterns or trends in the data, indicated disparity in the measurements due to the implementation of different techniques (Figure 3, Table 1).

Pairwise comparisons of the images acquired using DLIR and ASIR-V in the BGA (basal ganglia area) presented increases in the SNR of 46.7% (*p* < 0.01) and 59.5% (*p* < 0.01) for gray and white matter, respectively. Likewise, in the posterior cranial fossa, DLIR reconstructions accounted for higher SNR values. Specifically, the SNR increased by 54.2% (*p* < 0.01) in the gray matter of the cerebellum and 60.1% (*p* < 0.01) in the white matter. In the assessment of the ventricular system based on the ROI placed in the fourth ventricle of the brain, the SNR values were 51.2% (*p* < 0.01) higher in DLIR reconstruction compared to ASIR-V. The DLIR-acquired images of BGA showed a 57.6% increase in the CNR compared to the ASIR-V scans, while the PCF (posterior cranial fossa) scans achieved a 50.3% increase. The mean SNR and CNR measurements are presented in Table 2. Our analysis revealed a statistically significant (*p* < 0.01) image improvement in both BGA and PCF under DLIR utilization. Amongst the 109 assessed scans, the highest recorded Dose Length Product (DLP) was 1208.77 mGy*cm, while the lowest was 433.27 mGy*cm. The average DLP was 903.97 mGy*cm.

**Figure 4:**
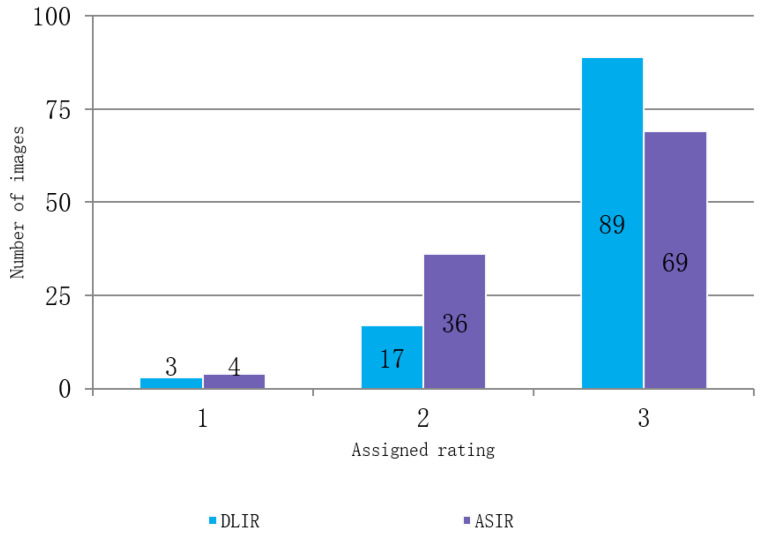
Subjective image quality assessment in DLIR and ASIR.

During the subjective assessment of the compared images, the researchers observed that DLIR had a slight advantage over ASIR-V in terms of its ability to visualize brain structures. A total of 89 of the acquired DLIR figures were given the highest possible mark (evaluated as being of excellent quality), compared to only 69 ASIR images. However, both reconstruction techniques faced difficulties in improving images of poor quality, with four of the ASIR, and three of the DLIR datasets rated 1. The average score awarded by the evaluators for the reconstructed images was 2.8 for DLIR and 2.6 for ASIR-V. The reviewers reached near-perfect agreement in the assessment of the images (Fleiss’ Kappa 0.88 and 0.91 for DLIR and ASIR-V, respectively). The subjective assessment is presented in Figure 4.

## 4. Discussion

The objective of this study was to conduct a comprehensive comparison of clinical brain CT image quality, utilizing both objective and subjective evaluation methods. Two distinct reconstruction algorithms, namely, ASIR-V and DLIR, were employed in this study. The primary advantage of our approach was the collection of average CT values within regions of interest (ROIs) for both protocols during a single scan. This methodology ensured the exclusion of any potential variations arising from changes in brain morphology or the emergence of previously undetected pathological findings. Additionally, obtaining both reconstructions in a single scan enabled the evaluation of image quality enhancement while maintaining a consistent radiation dose.

The results of our study demonstrate that DLIR provided a notable improvement in SNR, with increases of 46.68% up to 60.12% observed in both gray and white matter, as well as CNR increases of 57.58% and 50.29% in the BGA and PCF, respectively. The results of our study are in line with the current literature concerning the selection of patient cohorts, whether the studies implement comparable exclusion criteria, as in Kim et al.’s research [17], or administer no clinically valid exclusion criteria at all, as in the corresponding study of Alagic et al. [7]. However, our study contains a larger patient group. Moreover, in our assessment, a greater focus was placed on establishing PCF image quality improvement, whereas the previous studies aimed mostly to analyze the basal ganglia and cortex regions [7,17].

The utilization of DLIR in non-contrast head CT scans appears to have particular significance as differences in mean CT numbers between the grey and white matter of the brain are very subtle. Moreover, the neuralgic region of the cerebellum, pons, and medulla is rarely free of motion and bone-based artifacts, thus making image improvement and noise canceling in this region of high clinical importance [19,20]. Conventional ASIR-V approaches face challenges due to the increasing number of parameters, which can make convergence difficult. Recent studies have demonstrated that DLIR can handle intricate problems owing to training processes, thus surpassing the limitations of ASIR-V. DLIR was trained with thousands of FBP images that had been taken at both low and high dose levels, obtaining images free of statistical, physical, mechanical, or physiological biases. The high-dose FBP images were set as the target images for the DLIR recon algorithm, while the respective low-dose ones, and more specifically, their sinograms, fed the input layer of the algorithm. The neurons of the DLIR algorithm were finetuned in an iterative process, such that low-dose sinograms managed to achieve high dose level image quality [6].

There are several limitations to our study that should be acknowledged. As a result of the exclusion criteria we stated, this retrospective study had a relatively small sample size. Even though the number of patients in our study was larger compared to recent studies that compared DLIR and ASIR-V [7,17], it is necessary to conduct further research with a larger patient cohort to confirm our findings. Secondly, our study only included patients without clear neuropathological evidence or foreign bodies, and we were not able to assess the improvement in diagnostic accuracy. While our results indicate that DLIR yields superior image quality in individuals with normal-aging brains, additional studies involving patients with different clinical conditions are necessary to assess the impact of DLIR on lesion detection and diagnostic accuracy. Additionally, we only investigated CT images using a standard radiation dose protocol that was adjusted to our institution’s clinical requirements. Given that radiation dose reduction can be achieved by adjusting the reconstruction mechanism, it is important to assess the effects of DLIR on low-dose radiation protocols in future studies. Moreover, CNR and SNR are linear metrics, and as such, a more sophisticated image quality assessment in terms of detectability index (d’) and MTF (modulation transfer function) or TTF (task-based transfer function)/NPS-based metrics will follow.

In conclusion, our study demonstrates that DLIR surpasses ASIR-V in enhancing image quality and reducing noise in non-contrast head CT examinations. DLIR exhibited superiority in both subjective and objective assessments of image quality improvement. These findings suggest that DLIR has the potential to become the standard method for reconstructing head CT scans. However, further research is needed to validate these results, consider different patient populations, and explore the potential benefits of DLIR in low-dose radiation protocols using comprehensive image quality metrics.

## Figures and Tables

**Table 1:** Limits of agreement and bias for Bland–Altman plot assessment of DLIR and ASIR measurement similarity.

Region	Limits of Agreement		Mean Bias
ROI 1	−2.6483	1.8449	0.4017
ROI 2	−1.0265	1.1848	0.0791
ROI 3	−2.5678	2.0095	−0.2791
ROI 4	−3.0607	3.6421	0.2907
ROI 5	−1.3425	1.0463	−0.1480

**Table 2:** Objective image quality evaluation of head CT: DLIR compared to ASIR.

BGA					
	DLIR	ASIR	DLIR vs. ASIR	Mean Difference between DLIR and ASIR	*p*-Value
GM SNR	9.52 ± 2.12	6.49 ± 1.30	46.68%	3.03 ± 2.32	<0.001
WM SNR	7.48 ± 1.47	4.69 ± 1.03	59.49%	2.78 ± 1.88	<0.001
CNR	2.08 ± 0.58	1.32 ± 0.40	57.58%	0.76 ± 0.62	<0.001
**PCF**					
	**DLIR**	**ASIR**	**DLIR vs. ASIR**	**Mean difference between DLIR and ASIR**	***p*** **-value**
GM SNR	11.67 ± 2.32	7.57 ± 1.45	54.16%	4.10 ± 2.82	<0.001
WM SNR	7.83 ± 1.72	4.89 ± 1.11	60.12%	2.94 ± 2.06	<0.001
CNR	2.57 ± 0.70	1.71 ± 0.50	50.29%	0.86 ± 0.74	<0.001
**IV Ventricle**					
	**DLIR**	**ASIR**	**DLIR vs. ASIR**	**Mean difference between DLIR and ASIR**	***p*** **-value**
SNR	1.89 ± 0.45	1.25 ± 0.32	51.20%	0.63 ± 0.50	<0.001

BGA—basal ganglia area, DLIR—Deep Learning Image Reconstruction, ASIR—Adaptive Statistical Iterative Reconstruction, SNR—signal-to-noise ratio, GM—grey matter, WM—white matter, CNR—contrast-to-noise ratio.

## Data Availability

Restrictions apply to the availability of these data. Data generated or analyzed during this study are available from the corresponding author by request subject to institutional review and a data use agreement.
